# Supplementary material for: A therapeutic antibody targeting osteoprotegerin attenuates severe experimental pulmonary arterial hypertension
Source: Nat Commun. 2019 Nov 15;10:5183. doi: 10.1038/s41467-019-13139-9 (PMC6858314; doi:10.1038/s41467-019-13139-9)
Supplement: Supplementary file 1 — Supplementary Information [file 41467_2019_13139_MOESM1_ESM.pdf]

Supplementary Information

A therapeutic antibody targeting osteoprotegerin attenuates severe experimental pulmonary arterial hypertension

Arnold et al

Supplementary Methods

Homogeneous Time Resolved Fluorescence (HTRF) assay

The OPG/TRAIL HTRF assay was prepared using a starting concentration of 120 nM of each antibody sequentially diluted 1:3 to generate a 11-point curve (0.002 - 120 nM). 5 µL of each dilution was transferred to a white 384 well, low-volume, non-binding surface polystyrene plate (Greiner) and mixed with 5 µL of 4 nM human OPG-Fc prepared in HTRF assay buffer (PBS (Sigma) + 0.53 M KF (Sigma) + 0.1% w/v BSA (Sigma)). 5 µL of TRAIL diluted to 16 nM in HTRF buffer assay buffer were then added to assay plates with the exception of control wells used to detect non-specific binding. Before adding the detection solution, plates were incubated for 30 minutes at room temperature to allow receptor-ligand interaction to occur. 10 µL of a solution composed of anti-human Fc D2 (Cisbio) and anti-Flag cryptate (Cisbio) at 1/100 in HTRF assay buffer was added to 5 assay plates.

The HTRF assay to identify antibodies able to inhibit OPG/RANKL interaction was set up following the same method but using 20 nM hOPG-Fc (Seq ID No:154) mixed with 55.6 nM 647-RANKL. Detection solution was prepared using anti-Fc cryptate (Cisbio) diluted at 1/100 in HTRF assay buffer. Control curves were set up using anti-OPG antibody MAB805/Mouse IgG1 prepared at 120 nM working concentration in HMM and diluted 1:3, 11 dilution points (0.002 - 120 nM). Plates were incubated for 1 hour at room temperature and protected from light before measurement of wells at 620 nm and 665 nm emission wavelengths using an EnVision plate reader (Perkin Elmer). Data were analysed by calculating 665/620 ratio (Supplementary Equation 1) and % Specific Binding (Supplementary Equation 2) for each sample.

Supplementary Equation 1: Calculation of 665/620 ratio = (sample 665/620 nm value)

Supplementary Equation 2: Percentage of % TRAIL and RANKL specific binding Using 665/620 nm ratio (Supplementary Equation 1) (HTRF) % of specific binding = sample value - non-specific binding ×100 total binding - non-specific binding

Surface plasmon resonance (SPR)

Affinity of purified anti-OPG antibodies were assessed by label-free surface plasmon resonance (SPR). This analysis was carried out on the ProteOn XPR36 (BioRad) array SPR

machine. An anti-mouse IgG capture surface was created on a GLC biosensor 25 chip using amine coupling of an anti-mouse IgG (GE Healthcare). Test antibodies were captured on this surface and human, rat and cyno OPG (produced in-house, Kymab) were used as analyte. The assay was carried out at 25°C using HBS-EP (Teknova H8022). Buffer alone was used to reference the binding sensorgrams. The data was analysed using the 1:1 model inherent to the ProteOn XPR36 analysis software. All the affinity determinations were performed with purified hybridoma material so (human mouse chimera).

Supplementary Figures

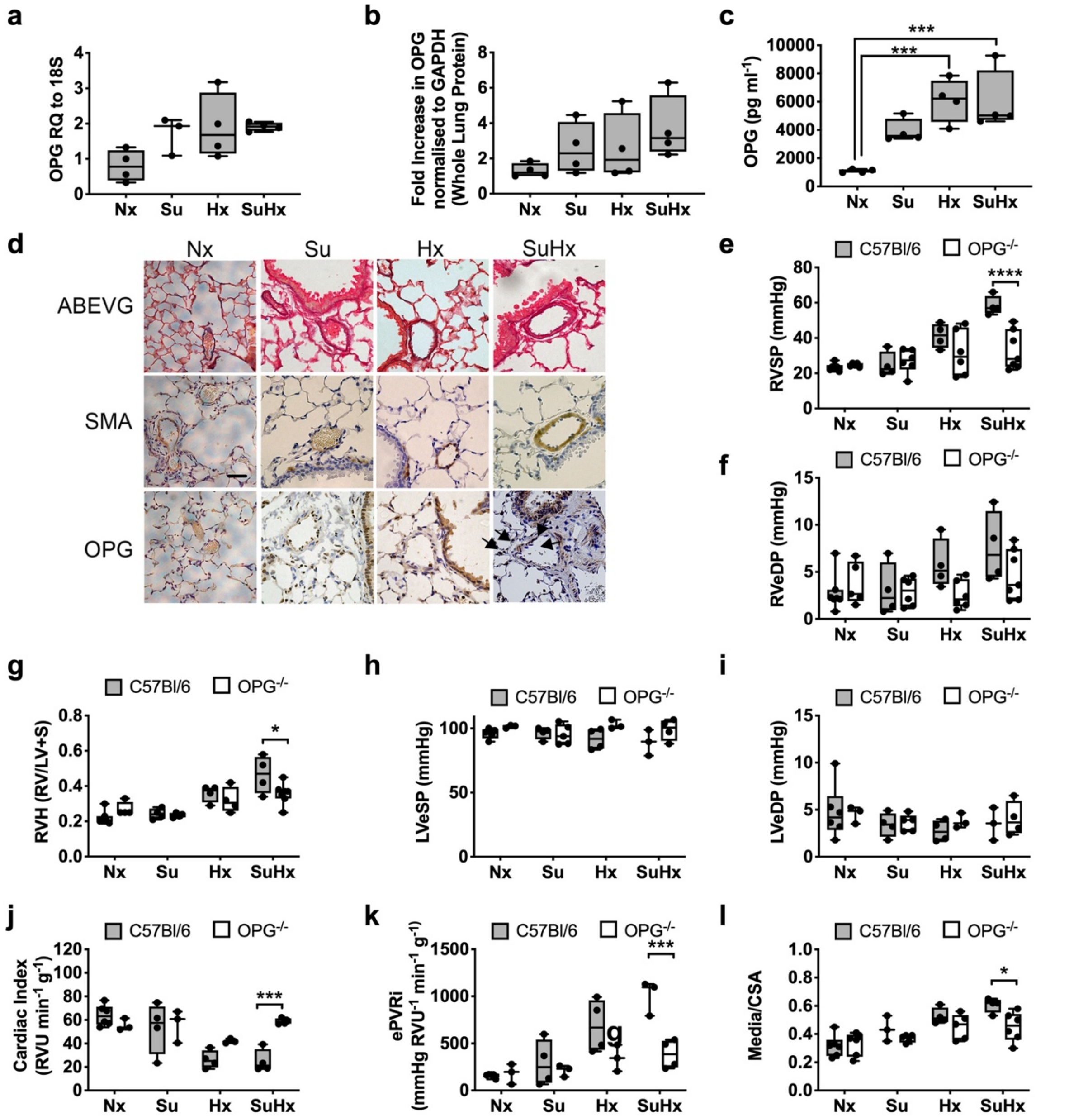

Supplementary Figure 1 - OPG expression in the SuHx mouse.

Box and Whisker graphs show the relative quantity of OPG transcript in whole lung tissue (a), whole lung OPG protein levels (b) and serum levels of OPG (c). Composite panel (d) shows representative photomicrographs of serial lung sections from normoxia (Nx), Sugen5416 (Su), hypoxia (Hx) and Sugen5416 plus hypoxia (SuHx) treated mice at 3 weeks. Sections were stained with Alcian Blue Elastic van Gieson (ABEVG) or immunostained for α-smooth muscle actin (α-SMA), or osteoprotegerin (OPG). OPG is present within the media and perivascular regions of remodelled small pulmonary arteries of SUHX mice. Box plot (e) shows right ventricular systolic pressure (RVSP), (f) right ventricular end-diastolic pressure, (g) right

ventricular hypertrophy (RVH), (h) left ventricular end-systolic pressure, (i) left ventricular end-diastolic pressure, (j) Cardiac Index, (k) estimated pulmonary vascular resistance index (ePVRi) and (l) the degree of medial wall thickness as a ratio of total vessel size (Media/CSA). Box and Whisker plots represent the interquartile range with the line representing the median, with the error bars showing the full range. Each animal is represented by a dot (C57 Nx (n=6), C57 Su (n=4), C57 Hx (n=4), C57 SuHx (n=4), $OPG^{-/-}$ Nx (n=5), $OPG^{-/-}$ Su (n=6), $OPG^{-/-}$ Hx (n=6), $OPG^{-/-}$ SuHx (n=7) animals per group). *=p<0.05, **=p<0.01, ***=p<0.001 compared to saline treated rats or normoxic mice using one-way ANOVA followed by Tukey's post-hoc test for multiple comparison. All images are representative of group at each time point and are presented at their original magnification x400, scale bars represent 50 µm. Source data are provided as a Source Data file.

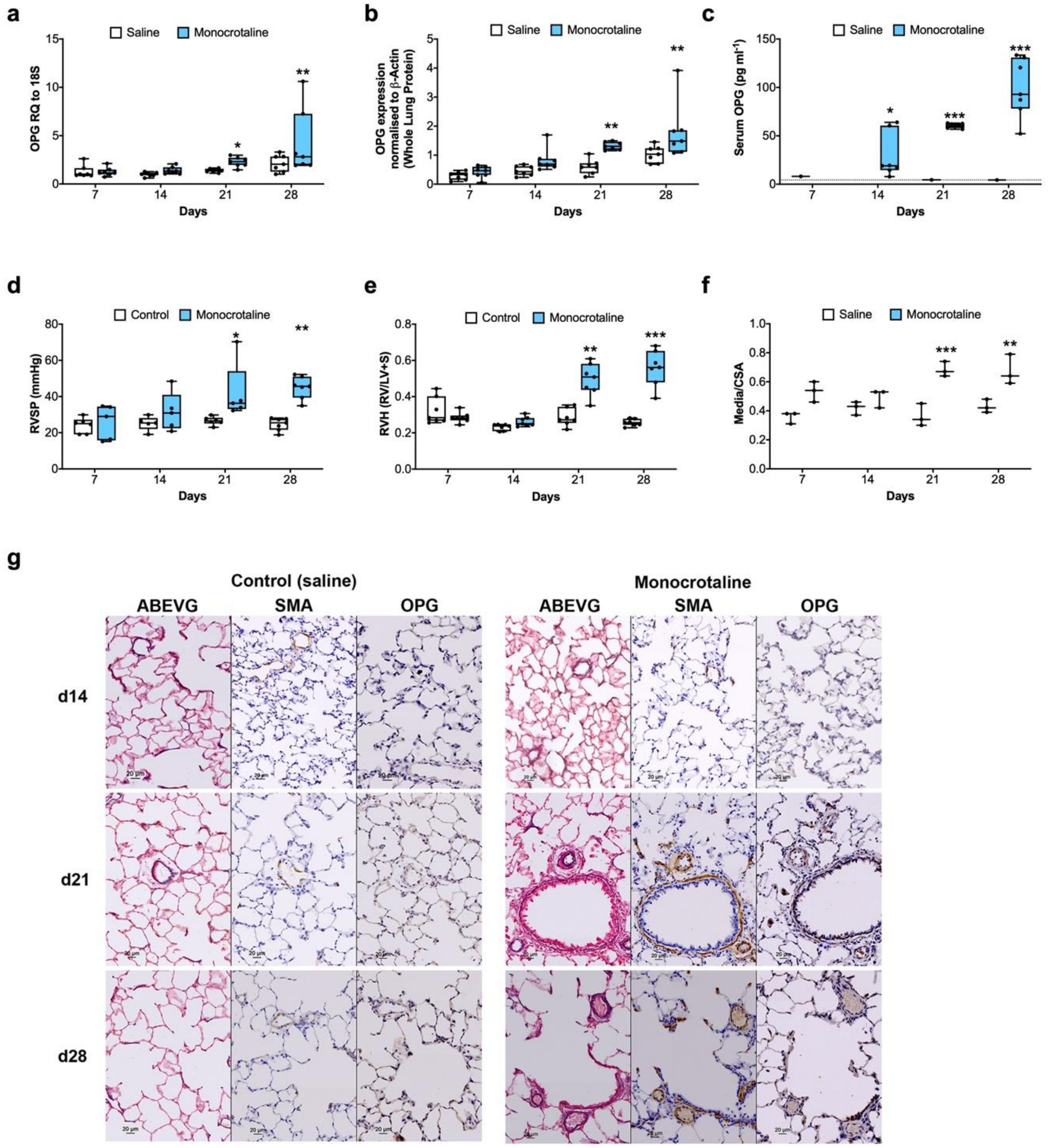

Supplementary Figure 2 – OPG expression in the Mct Rat.

Box and whisker plots show the relative quantity of OPG transcript in whole lung tissue (a), whole lung OPG protein levels (b) and serum levels of OPG (c). Panel (d) shows right ventricular systolic pressure (RVSP), (e) right ventricular hypertrophy (RVH), (f) the degree of medial wall thickness as a ratio of total vessel size (Media/CSA). Panel (g) shows representative photomicrographs of serial lung sections from control saline and monocrotaline (Mct) injected rats at 14, 21 and 28 days post injection. Sections were stained with Alcian Blue Elastic van Gieson (ABEVG) or immunostained for α-smooth muscle actin (α-SMA), or osteoprotegerin (OPG). OPG is present within the media and peri-vascular regions of remodelled small pulmonary arteries of Mct rats increasing between d14-28. Box and Whisker

plots represent the interquartile range with the line representing the median and the whiskers showing the full range of the data. Each animal is represented by a dot; for panels a-c &e n=7 animals per group, and for panel d n=6. *=p<0.05, **=p<0.01, ***=p<0.001 compared to saline treated rats using two-way ANOVA followed by Tukey's post-hoc analysis for multiple comparison. All images are representative of group at each time point and are presented at their original magnification x400, scale bars represent 50 µm. Source data are provided as a Source Data file.

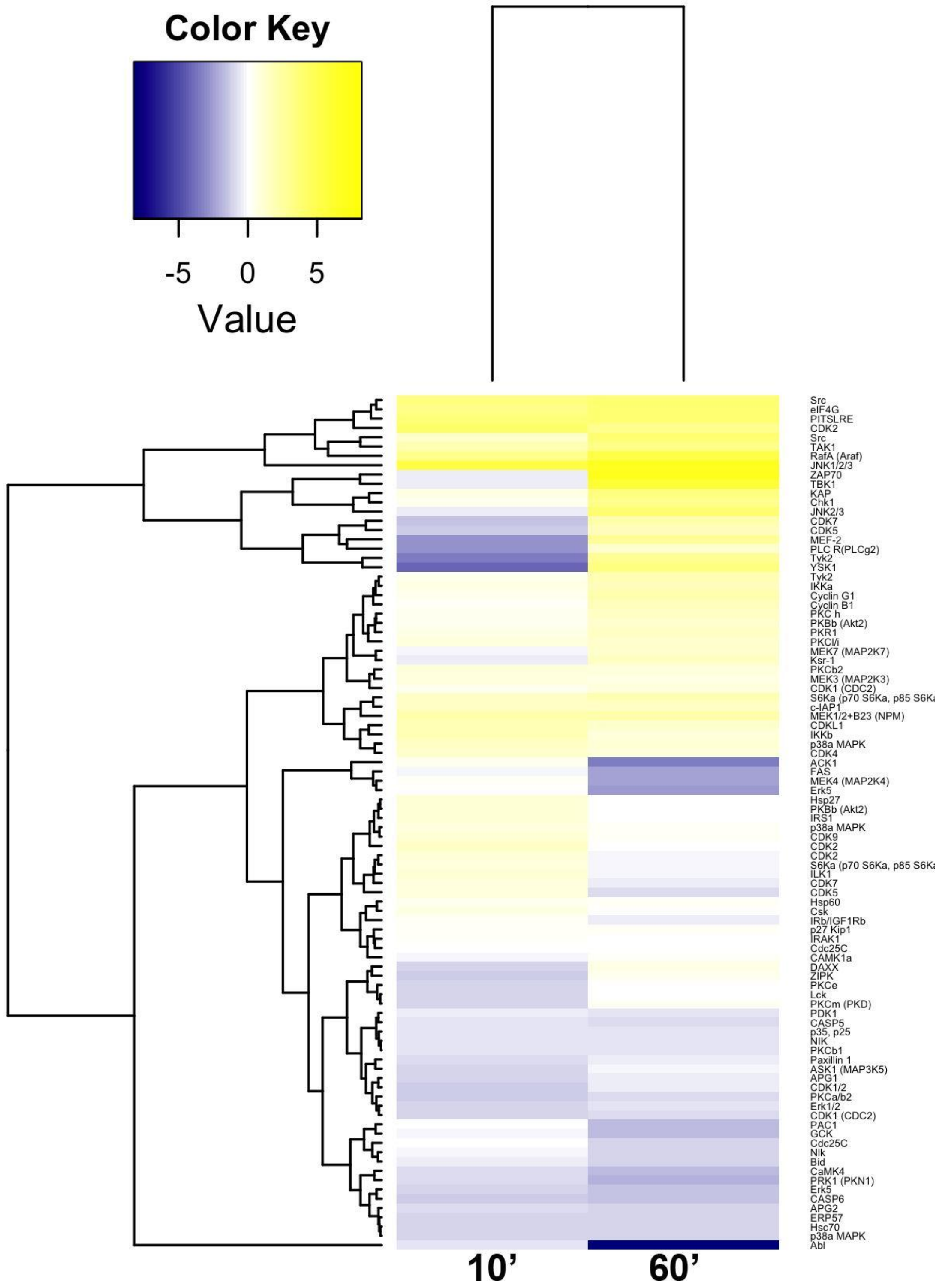

Supplementary Figure 3 - Intracellular signalling induced by OPG in PASMC.

Heat map demonstrates 63 from 800 phosphorylation and pan-specific antibodies that were significantly regulated by OPG (50 ng ml^{-1}) at either 10, 60 min as a ratio to unstimulated cells by KinexTM antibody microarray (KAM) n=4, from pulled triplicate donors. Source data are provided as a Source Data file.

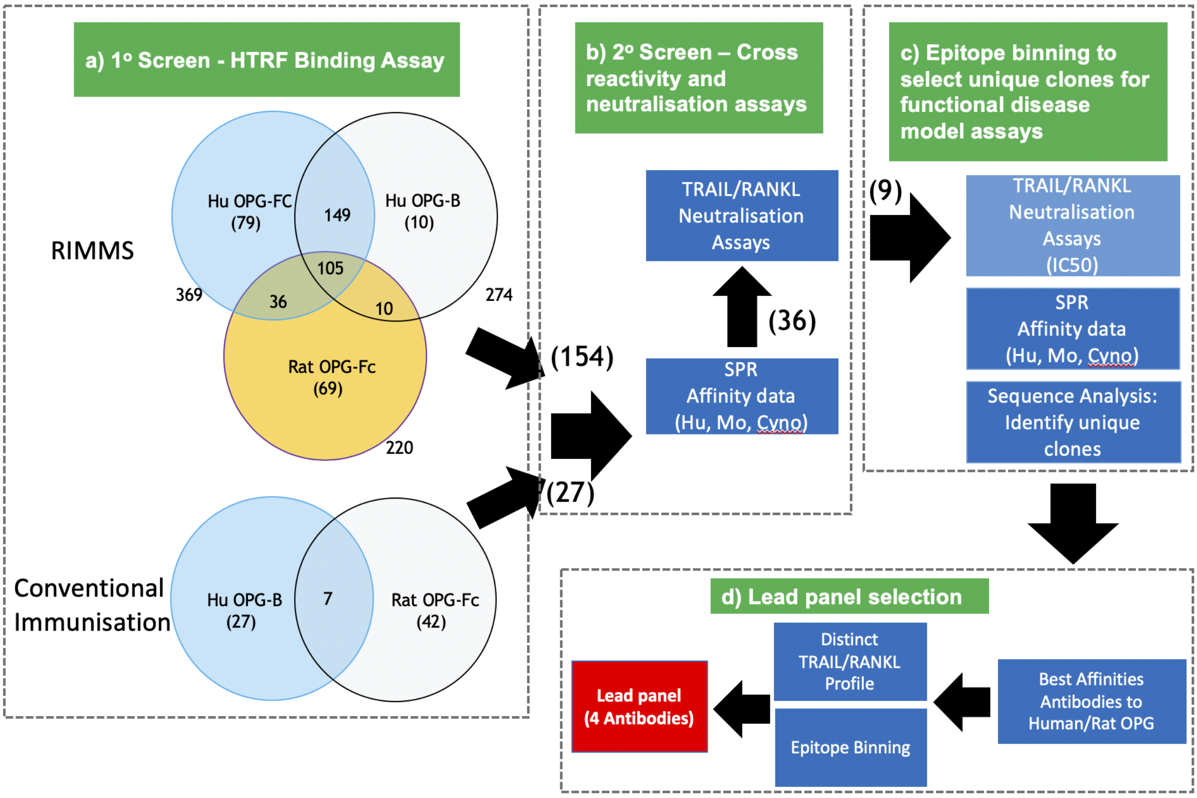

e) Lead Panel of anti-OPG antibodies for phenotype screens

Antibody	Kd (nM) Human OPG	Kd (nM) Rat OPG	Kd (nM) Cyno OPG	Epitope Binning	OPG- Trail inhibition	OPG- RANKL inhibition
**KY-1**	0.17	1.35	14.8	1	Inhibition Max Specific binding = 4.8%	No Inhibition
**KY-2**	0.1	7.39	CNROR	2	Inhibition Max Specific binding = 20 %	Partial Inhibition Max Specific Binding =47.1%
**KY-3**	CNROR	0.12	CNROR	3	Complete Inhibition IC_{50} 1.4nM	Inhibition Max Specific binding = 27.1 %
**KY-4**	0.18	1.3	0.166	4	Partial Inhibition Max Specific Binding =50.7%	Partial Inhibition Max Specific Binding = 65.1 %

Supplementary Figure 4 - Screening for lead candidate human anti-OPG therapeutic monoclonal antibodies.

(a) Conventional intraperitoneal injections as well as a rapid immunisation at multiple sites (RIMMS) immunisation regimes were set up using human and rat recombinant OPG protein as immunogen using the KyMouse™[25]. (b) A secondary screen identified high affinity antibodies which were then tested for their neutralisation profiles for the interaction of OPG with TRAIL and RANKL. (c) Epitope binning was then performed and the lead panel of antibodies chosen (d) based on the best affinity antibody identified from each epitope bin. (e) summary of binding parameters and neutralisation profiles for the 4 lead antibodies as identified in a single experiment using purified chimeric hybridoma antibodies. nM = nano molar, CNROR = could not resolve off-rate

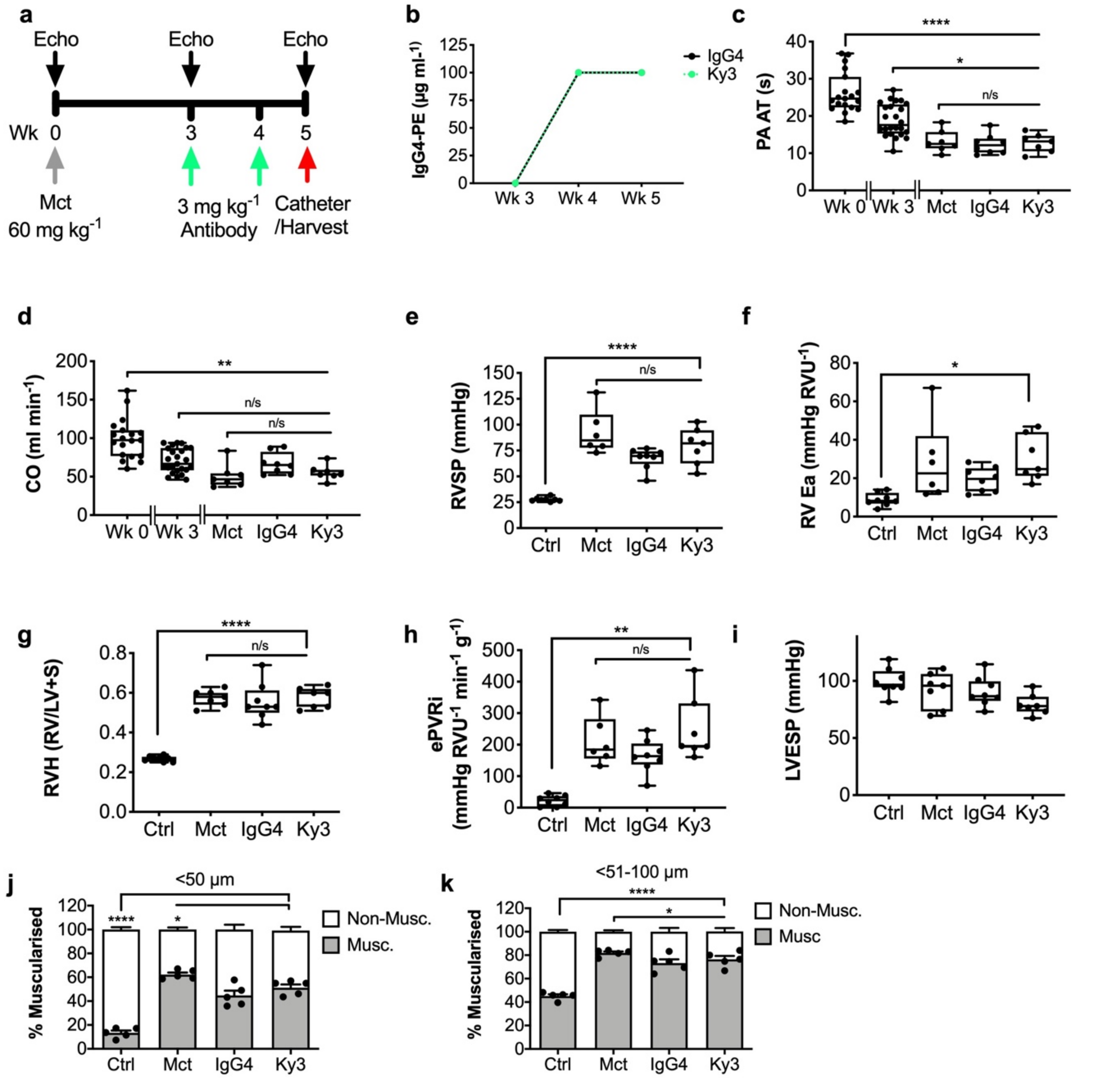

Supplementary Figure 5 - Therapeutic delivery Ky3 to severe Mct induced PAH in rats.

Panel (a) shows a schema demonstrating the disease and treatment time course. (b) Plasma concentrations of antibody and IgG. Bar graphs show (c) Pulmonary Artery Acceleration Time (PA AT), (d) cardiac output, (e) right ventricular systolic pressure (RVSP), (f) right ventricular arterial elastance (RV Ea), (g) right ventricular hypertrophy (RVH), (h) estimated pulmonary vascular resistance (ePVRi), (i) left ventricular end-systolic pressure (LVESP). Bar graphs (j) show the relative percentage of muscularised small pulmonary arteries and arterioles in <50 µm vessels and (k) 51-100 µm vessels. Box and Whisker plots represent the interquartile range with the line representing the median and the whiskers the full range of the data; dots represent individual animals. (Ctrl n=8, Mct=6, IgG4 n=8, Ky3 n=7 animals per group), *=p<0.05,

**=$p<0.01$, ***=$p<0.001$ compared to IgG treated rats using one-way ANOVA followed by Tukey’s post-hoc test for multiple comparison. Source data are provided as a Source Data file.
