# Supplementary material for: A therapeutic antibody targeting osteoprotegerin attenuates severe experimental pulmonary arterial hypertension
Source: Nat Commun. 2019 Nov 15;10:5183. doi: 10.1038/s41467-019-13139-9 (PMC6858314; doi:10.1038/s41467-019-13139-9)
Supplement: Supplementary file 3 — Reporting Summary [file 41467_2019_13139_MOESM3_ESM.pdf]

natureresearch

Corresponding author(s): Lawrie, Allan

Last updated by author(s): Sep 27, 2019

Reporting Summary

Nature Research wishes to improve the reproducibility of the work that we publish. This form provides structure for consistency and transparency in reporting. For further information on Nature Research policies, see Authors & Referees and the Editorial Policy Checklist .

Statistics

For all statistical analyses, confirm that the following items are present in the figure legend, table legend, main text, or Methods section.

n/a	Confirmed	
☐	☒	The exact sample size (*n*) for each experimental group/condition, given as a discrete number and unit of measurement
☐	☒	A statement on whether measurements were taken from distinct samples or whether the same sample was measured repeatedly
☐	☒	The statistical test(s) used AND whether they are one- or two-sided<br>*Only common tests should be described solely by name; describe more complex techniques in the Methods section.*
☒	☐	A description of all covariates tested
☐	☒	A description of any assumptions or corrections, such as tests of normality and adjustment for multiple comparisons
☐	☒	A full description of the statistical parameters including central tendency (e.g. means) or other basic estimates (e.g. regression coefficient) AND variation (e.g. standard deviation) or associated estimates of uncertainty (e.g. confidence intervals)
☒	☐	For null hypothesis testing, the test statistic (e.g. *F*, *t*, *r*) with confidence intervals, effect sizes, degrees of freedom and *P* value noted<br>*Give P values as exact values whenever suitable.*
☒	☐	For Bayesian analysis, information on the choice of priors and Markov chain Monte Carlo settings
☒	☐	For hierarchical and complex designs, identification of the appropriate level for tests and full reporting of outcomes
☒	☐	Estimates of effect sizes (e.g. Cohen's *d*, Pearson's *r*), indicating how they were calculated

Our web collection on statistics for biologists contains articles on many of the points above.

Software and code

Policy information about availability of computer code

Data collection	*Provide a description of all commercial, open source and custom code used to collect the data in this study, specifying the version used OR state that no software was used.*
Data analysis	*Provide a description of all commercial, open source and custom code used to analyse the data in this study, specifying the version used OR state that no software was used.*

For manuscripts utilizing custom algorithms or software that are central to the research but not yet described in published literature, software must be made available to editors/reviewers. We strongly encourage code deposition in a community repository (e.g. GitHub). See the Nature Research guidelines for submitting code & software for further information.

Data

Policy information about availability of data

All manuscripts must include a data availability statement. This statement should provide the following information, where applicable:

- Accession codes, unique identifiers, or web links for publicly available datasets
- A list of figures that have associated raw data
- A description of any restrictions on data availability

The data that support the findings of this study are available from the corresponding author upon reasonable request.

Field-specific reporting

Please select the one below that is the best fit for your research. If you are not sure, read the appropriate sections before making your selection.

Life sciences study design

All studies must disclose on these points even when the disclosure is negative.

Sample size	Sample size was calculated using in house data obtained from previous studies. A sample size of at least 4 animals was used to provide greater than 95% power to detect a difference in RVSP of 10 mmHg with a SD of 3 mmHg with 95% confidence. Additional animals were studied in large group comparisons and to obtain sufficient tissue for analysis and allow for multiple comparison testing.
Data exclusions	No data were excluded from the study.
Replication	The therapeutic efficacy of the Ky3 antibody was tested using antibody derived from 2 separate manufacturing batches in greater than 3 distinct studies.
Randomization	Animals were randomised to treatment regime and investigators blinded to treatment labels
Blinding	Investigators were blinded to treatment groups. All animals were assigned a unique ID and phenotype recorded in a bespoke research database. Sheffield investigators were blinded to antibody labels until after all the phenotype data were collected.

Reporting for specific materials, systems and methods

We require information from authors about some types of materials, experimental systems and methods used in many studies. Here, indicate whether each material, system or method listed is relevant to your study. If you are not sure if a list item applies to your research, read the appropriate section before selecting a response.

Materials & experimental systems

n/a	Involved in the study	
☐	☒	Antibodies
☒	☐	Eukaryotic cell lines
☒	☐	Palaeontology
☐	☒	Animals and other organisms
☐	☒	Human research participants
☒	☐	Clinical data

Methods

n/a	Involved in the study	
☒	☐	ChIP-seq
☒	☐	Flow cytometry
☒	☐	MRI-based neuroimaging

Antibodies

Antibodies used	antibody manufacturer and catalogue numbers are referenced in the manuscript
Validation	All antibodies were used in accordance with the manufacturers instructions and guidelines on their website, positive control tissues were used where possible and secondary only controls were used.

Animals and other organisms

Policy information about studies involving animals; ARRIVE guidelines recommended for reporting animal research

Laboratory animals	Reported in the methods section of the manuscript
Wild animals	N/A
Field-collected samples	N/A
Ethics oversight	UK Home office License and Institution Protocol approval as stated in manuscript methods

Note that full information on the approval of the study protocol must also be provided in the manuscript.

Human research participants

Policy information about studies involving human research participants

Population characteristics	Lung tissue samples (n=12); 4 male, 8 female, mean age 40 +/- 11 from patients with PAH; and from control subjects undergoing lung resection for cancer or from unused donor-lung (n=6; 4 male, 2 female, mean age 55 +/- 21)
Recruitment	Patients were recruited from those undergoing lung transplant at Royal Papworth Hospital NHS Foundation Trust. Informed consent was obtained from all patients who contributed samples to the study.

Ethics oversight | All samples were collected and distributed under the Royal Papworth Hospital NHS Trust Tissue Bank (PHRTB) Ethics.

Note that full information on the approval of the study protocol must also be provided in the manuscript.
